# Association of vitamin D with risk of type 2 diabetes: A Mendelian randomisation study in European and Chinese adults

**DOI:** 10.1371/journal.pmed.1002566

**Published:** 2018-05-02

**Authors:** Ling Lu, Derrick A. Bennett, Iona Y. Millwood, Sarah Parish, Mark I. McCarthy, Anubha Mahajan, Xu Lin, Fiona Bragg, Yu Guo, Michael V. Holmes, Shoaib Afzal, Børge G. Nordestgaard, Zheng Bian, Michael Hill, Robin G. Walters, Liming Li, Zhengming Chen, Robert Clarke

**Affiliations:** 1 Clinical Trial Service Unit and Epidemiological Studies Unit, Nuffield Department of Population Health, University of Oxford, Oxford, United Kingdom; 2 Laboratory of Nutrition and Metabolism, Institute for Nutritional Sciences, Shanghai Institutes for Biological Sciences, Chinese Academy of Sciences, Shanghai, China; 3 Medical Research Council Population Health Research Unit at the University of Oxford, Oxford, United Kingdom; 4 Oxford Centre for Diabetes, Endocrinology and Metabolism, University of Oxford, Oxford, United Kingdom; 5 Wellcome Trust Centre for Human Genetics, Nuffield Department of Medicine, University of Oxford, Oxford, United Kingdom; 6 Chinese Academy of Medical Sciences, Dong Cheng District, Beijing, China; 7 Department of Clinical Biochemistry, Herlev and Gentofte Hospital, Copenhagen University Hospital, Herlev, Denmark; 8 Faculty of Health and Medical Sciences, University of Copenhagen, Copenhagen, Denmark; 9 Department of Epidemiology and Biostatistics, Peking University Health Science Center, Peking University, Beijing, China; King’s College London, UNITED KINGDOM

## Abstract

**Background:**

Observational studies have reported that higher plasma 25-hydroxyvitamin D (25[OH]D) concentrations are associated with lower risks of diabetes, but it is unclear if these associations are causal. The aim of this study was to test the relevance of 25(OH)D for type 2 diabetes using genetically instrumented differences in plasma 25(OH)D concentrations.

**Methods and findings:**

Data were available on four 25(OH)D single nucleotide polymorphisms (SNPs; *n* = 82,464), plasma 25(OH)D concentrations (*n* = 13,565), and cases with diabetes (*n* = 5,565) in the China Kadoorie Biobank (CKB). The effects on risk of diabetes were assessed by a genetic score using two 25(OH)D synthesis SNPs (*DHCR7*-rs12785878 and *CYP2R1*-rs10741657), with and without the addition of SNPs affecting the transport (*GC/DBP*-rs2282679) and catabolism (*CYP24A1*-rs6013897) of 25(OH)D. The CKB results were combined in a meta-analysis of 10 studies for the 2 synthesis SNPs (*n* = 58,312 cases) and 7 studies for all 4 SNPs (*n* = 32,796 cases). Mean (SD) 25(OH)D concentration was 62 (20) nmol/l in CKB, and the per allele effects of genetic scores on 25(OH)D were 2.87 (SE 0.39) for the synthesis SNPs and 3.54 (SE 0.32) for all SNPs. A 25-nmol/l higher biochemically measured 25(OH)D was associated with a 9% (95% CI: 0%–18%) lower risk of diabetes in CKB. In a meta-analysis of all studies, a 25-nmol/l higher genetically instrumented 25(OH)D concentration was associated with a 14% (95% CI: 3%–23%) lower risk of diabetes (*p* = 0.01) using the 2 synthesis SNPs. An equivalent difference in 25(OH)D using a genetic score with 4 SNPs was not significantly associated with diabetes (odds ratio 8%, 95% CI: −1% to 16%, lower risk, *p* = 0.07), but had some evidence of pleiotropy. A limitation of the meta-analysis was the access only to study level rather than individual level data.

**Conclusions:**

The concordant risks of diabetes for biochemically measured and genetically instrumented differences in 25(OH)D using synthesis SNPs provide evidence for a causal effect of higher 25(OH)D for prevention of diabetes.

## Abstract 2

Robert Clarke and colleagues, using a Mendelian Randomisation approach, reveal a link between higher levels of vitamin D and type 2 diabetes prevention, based on data from 2 large cohorts.

## Author summary

**Why was this study done?:**

- There is a debate about whether low vitamin D status increases the risk of diabetes or is just a marker of overall poor health. Observational studies have demonstrated that individuals with higher vitamin D status in European populations have lower risks of developing type 2 diabetes.

**What did the researchers do and find?:**

- The present report, involving approximately 82,500 adults living in China, replicated the association of higher vitamin D status with lower risk of type 2 diabetes in Chinese adults.
- A combined analysis of over 58,000 cases and 370,000 controls in European and Chinese adults demonstrated that genetically instrumented increases in a circulating marker of vitamin D status, corresponding to increases achievable by vitamin D supplements, were associated with a 14% lower risk of diabetes.

**What do these findings mean?:**

- The concordant results of both the observational studies and the genetic studies indicate that higher vitamin D status is associated with lower risk of diabetes and provide support for a causally protective effect of higher vitamin D status for prevention of type 2 diabetes.

## Introduction

The incidence of type 2 diabetes has increased substantially in both high-income and low- and middle-income countries in recent decades [1]. Vitamin D insufficiency, defined as plasma 25-hydroxyvitamin D (25[OH]D) concentration < 75 nmol/l, is common in European and Asian populations, particularly among those living at high latitude, during the winter months, or among those in cities with poor air quality [2–4]. Observational studies have reported that higher plasma 25(OH)D concentrations are associated with lower risks of diabetes [5–7]. A meta-analysis of 22 prospective studies of individuals of European descent, involving 8,492 cases of diabetes [5], reported that a 25-nmol/l higher 25(OH)D concentration was associated with a 17% lower risk of diabetes, but the causal relevance of this association is uncertain. Previous randomised trials have reported conflicting results for the effects of supplementation with vitamin D on risk of diabetes, but these trials may not have been large enough or used sufficient doses of vitamin D to detect a benefit [8–12].

Mendelian randomisation (MR) studies of genetic variants can help to assess the causal relevance of vitamin D status for risk of diabetes. Since genotypes are randomly assigned before birth, associations of genetic variants with risk of diabetes are not constrained by confounding or reverse causality, which limit the interpretation of observational studies [13]. However, the validity of MR studies is dependent on the effects of the genetic variants on risk of diabetes being mediated solely via their effects on plasma 25(OH)D concentration and not by some other trait (pleiotropy) [13]. Genome-wide association studies have identified 4 single nucleotide polymorphisms (SNPs) that influence plasma 25(OH)D concentration (S1 Fig) [14,15]. The genetic variants affecting the synthesis of 25(OH)D, in the genes *DHCR7* (encoding 7-dehydrocholesterol reductase enzyme for synthesis of pre-vitamin D3) and *CYP2R1* (encoding the 25-hydroxylase enzyme for conversion of vitamin D3 to 25[OH]D), are upstream of 25(OH)D, and these are not known to have pleiotropic effects (S1 Fig) [13]. In contrast, 2 other genetic variants, in the genes *GC/DBP* (the group-specific component that encodes vitamin D binding protein) and *CYP24A1* (which encodes the 24-hydroxylase involved in the clearance of 25[OH]D), have potential pleiotropic effects (S1 Fig) [13]. The genetic variants involved in vitamin D binding protein cause discrepant effects on free versus total 25(OH)D concentration [16,17], and variants affecting the catabolism of vitamin D (*CYP24A1*) also influence plasma concentrations of phosphate and FGF-23 [18], such that these genetic variants may affect diabetes independent of their effects on 25(OH)D concentration.

Previous MR studies of 25(OH)D and type 2 diabetes have reported conflicting results [5,19–23], with studies using the 2 synthesis SNPs suggesting a possible protective effect [21], but studies using all 4 SNPs influencing 25(OH)D concentration reporting no association with diabetes [5,22]. Since the effects of these SNPs on plasma 25(OH)D concentrations were typically only about 3–3.5 nmol/l per allele, MR studies require a large number of cases to assess causality.

The aims of the present study were as follows: (i) to examine the associations of genetic scores for 25(OH)D concentration with the 2 synthesis SNPs (*DHCR7*-rs12785878 and *CYP2R1*-rs10741657) versus all available SNPs (2 synthesis SNPs in addition to 1 transport SNP [*GC/DBP*-rs2282679] and 1 catabolism SNP [*CYP24A1*-rs6013897]) in 82,464 Chinese adults from the China Kadoorie Biobank (CKB) [24]; (ii) to conduct an updated meta-analysis of all genetic studies assessing the effects of genetically instrumented differences in plasma 25(OH)D concentrations on risk of type 2 diabetes in a primary analysis using the 2 synthesis SNPs and a secondary analysis using all 4 SNPs for 25(OH)D concentration; and (iii) to compare the risks of diabetes associated with equivalent differences in biochemically measured versus genetically instrumented plasma 25(OH)D concentrations.

## Methods

### CKB study population

Details of the CKB study design, methods, and participants have been previously reported [24] (S1 Table). The 512,891 participants were enrolled from 10 geographically diverse urban and rural areas in China. Participants aged 30–79 years were identified through local residential records and invited to attend survey clinics (~30% responded) [24]. The baseline survey was conducted between June 2004 and July 2008 and included data on demographic and lifestyle factors, medical history, and use of medication (see http://www.ckbiobank.org for details of questionnaire used in baseline survey). Physical measurements included blood pressure, weight, height, body mass index (BMI), waist—hip ratio (WHR), and body fat percentage. A 10-ml non-fasting venous blood sample was collected (with time of last meal recorded), and random plasma glucose was measured on site (Johnson & Johnson SureStep Plus, LifeScan). Ethics approval was obtained from the relevant local, national, and international ethics committees, and all participants provided written informed consent.

### Assays of plasma 25(OH)D concentration in CKB

After centrifugation of thawed stored EDTA plasma samples, plasma 25(OH)D concentration was measured using a Beckman Coulter Access 2 immunoassay in 13,565 participants (of whom 3,014 also participated in the genetic study). The laboratory participated in an international DEQAS scheme for 25(OH)D and had a mean (SD) bias of −11.8% (7.5%) from the target mean value during the analysis of the CKB study.

### Observational association of 25(OH)D concentration with diabetes in CKB

Participants answering “yes” to the question, “Has a doctor ever told you that you had diabetes?” at baseline were defined as having prevalent diabetes. Incident cases of diabetes were identified by electronic linkage to disease registries and national health insurance databases for hospitalisations due to diabetes (using International Classification of Diseases classification codes E10–E14 or use of specific antidiabetic medication). The present analysis included all incident cases occurring after baseline and prior to 1 January 2014. (Detailed review of medical records of 1,000 randomly selected incident cases of diabetes indicated a positive predictive value of 97% based on American Diabetes Association diagnostic criteria; see http://www.ckbiobank.org). After excluding the 496 individuals with prevalent diabetes at baseline (to avoid reverse causality), the observational association of plasma 25(OH)D concentration with incident type 2 diabetes was assessed with 979 cases among 13,069 individuals with plasma 25(OH)D concentration in CKB.

### SNP selection and genotyping in CKB

A total of 95,680 randomly selected CKB participants were genotyped using a panel of 384 SNPs (Illumina GoldenGate) (S2 Table). The genotyping panel included 2 synthesis SNPs (*DHCR7*-rs12785878 and *CYP2R1*-rs10741657), 1 transport SNP (*GC/DBP*-rs2282679), and 1 catabolism SNP (*CYP24A1*-rs6013897), identified as significantly associated with plasma 25(OH)D concentration in previous genome-wide studies [14]. The genotyping concordance was >99.9% for 2,063 pairs of sample replicates, and the genotyping success rate was 99.9% for each SNP. Samples with genotype call rate < 98%, excessive heterozygosity, gender mismatch between reported and genetically inferred gender, missing genotype data for relevant SNPs, or other potential linkage errors were excluded, as were first-degree relatives of included participants, leaving 82,464 individuals for genetic analysis. All genetic analyses in CKB included both prevalent and incident cases of diabetes.

### Meta-analysis of all genetic studies

Using the search terms “genetic studies of plasma 25(OH)D concentrations” or “genetic studies of vitamin D” or “diabetes”, we sought to identify further genetic studies, in addition to those included in a previous meta-analysis in 2015, for 25(OH)D and risk of diabetes using the PubMed and Web of Science databases [5]. Studies were restricted to those involving relevant genetic variants for 25(OH)D and at least 500 cases with diabetes. Five published reports [5,19–22] were identified (the EPIC-Germany [19], Tromsø [20], Copenhagen [21], and EPIC-Norfolk [22] studies and a meta-analysis of multiple European studies [5] [CCCS, ADDITION-Ely, Norfolk Diabetes, EPIC-InterAct, and DIAGRAM]; S2 and S3 Figs; S2 Table). The EPIC-Germany study [19] and the EPIC-Norfolk study [22] were excluded as they had been previously included in EPIC-InterAct. The Tromsø study was excluded because of missing data on the genotype distributions [20]. Additional unpublished data were obtained from the CKB study, the UK Biobank resource, the Copenhagen study [21], and non-overlapping individuals in the T2D Exome consortium (a meta-analysis of 40,723 non-overlapping individuals among 79,854 T2D case—control samples genotyped using the exome array for the DIAGRAM and T2D-GENES Consortium; S3 Table; S3 Fig). All 10 studies (UK Biobank, Norfolk Diabetes, DIAGRAM, Copenhagen, CCCS, ADDITION-Ely, T2D Exome consortium, EPIC-InterAct-metabochip, EPIC-InterAct-gwas, and CKB) included data on both synthesis SNPs and the transport SNP *(GC/DBP*), whereas only 7 of the studies had the catabolism SNP *(CYP24A1*).

### Statistical methods

#### Pre-specified analysis plan

It was pre-specified to first conduct an observational analysis of the association of plasma 25(OH)D with risk of diabetes in CKB, and assess associations of genetic variants with 25(OH)D and with diabetes in CKB, and then subsequently conduct an updated meta-analysis of the worldwide studies of genetic variants and risk of diabetes. The primary analyses were pre-specified to investigate the effects of the synthesis SNPs on risk of diabetes, and the secondary analyses assessed the effects of all four 25(OH)D SNPs on risk of diabetes. Pleiotropy was investigated for all 4 SNPs available in 7 studies and the 3 SNPs available in all 10 studies. Lastly, the analyses then compared the risks of diabetes associated with equivalent differences in biochemically measured and genetically instrumented 25(OH)D concentrations in the worldwide studies.

#### Observational analysis in CKB

Logistic regression analysis was used to estimate log odds ratios (ORs) and 95% confidence intervals (CIs) of incident diabetes for tertiles (or per 25-nmol/l higher 25[OH]D) after adjustment for age, sex, latitude, systolic blood pressure (SBP), physical activity, and percent body fat. The 95% CI for the OR for each tertile was plotted using floating absolute risks by estimation of the variance of the log risk, so that each tertile of 25(OH)D was accompanied by a 95% CI derived from the variance of the log risk for that tertile [25].

#### Genetic analyses in CKB

Genotype distributions of each SNP and deviation from Hardy—Weinberg equilibrium were assessed separately in each of the 10 geographical areas. Linear regression was used to assess the per allele effect of each SNP on plasma 25(OH)D concentration in the subset of 3,014 individuals with plasma 25(OH)D concentrations using 10-fold cross-validation to estimate valid internal weights [26]. Genetic scores were estimated for the 2 synthesis SNPs and all four 25(OH)D SNPs. The per allele effects on plasma 25(OH)D concentrations of the specified combinations of SNPs were used to construct the respective weighted genetic scores for 25(OH)D. The F-statistic was used to estimate the strength of the association of each SNP with 25(OH)D concentration, and F-statistic values > 10 were considered strong [26].

Linear regression was also used to assess the associations of each SNP with SBP, diastolic blood pressure (DBP), BMI, WHR, percent body fat, and random plasma glucose. All the genetic analyses were conducted separately for each area after adjusting for age at baseline, sex, and season, and were subsequently combined using inverse-variance-weighted meta-analysis.

Logistic regression was used to assess associations of individual SNPs and genetic scores with diabetes, separately for each area, and subsequently analyses were combined using inverse-variance-weighted meta-analysis. The per allele effect of each SNP on plasma 25(OH)D concentration in CKB was expressed as the difference in 25(OH)D concentration per copy of the 25(OH)D-raising allele. Instrumental variable analysis was used to estimate the causal effects of differences in genetically instrumented higher plasma 25(OH)D concentrations on risk of diabetes as previously described [26].

#### Updated meta-analysis of all genetic studies

The log ORs for diabetes per 25(OH)D-increasing allele and their standard errors for all SNPs were extracted from all the identified studies for the meta-analysis as previously described [26]. The log ORs for the SNPs and for the genetic scores combining the SNPs for risk of diabetes were scaled to 25-nmol/l higher 25(OH)D concentrations in all studies [26]. The effects of individual SNPs and the genetic scores on risk of diabetes in individual studies were combined in a meta-analysis by inverse-variance weighting using a fixed-effects model. Additional sensitivity analyses included combinations of (i) the lead synthesis and transport SNPs with the maximum effect on 25(OH)D concentration and (ii) both synthesis SNPs and the lead catabolism SNP. The per allele effects in all studies were weighted by the effect of each SNP on 25(OH)D concentration. Additional sensitivity analyses were conducted for the 3-SNP and 4-SNP genetic scores to assess the effects of pleiotropy using the MR—Egger regression method (where the *p*-value of the intercept is a valid test of directional pleiotropy) and the weighted median MR method [27]. All the statistical analyses were conducted using SAS version 9.2 and R version 3.01, and all reported *p*-values were nominal and 2-sided.

## Results

### Characteristics of the CKB population

Among the 82,464 CKB participants in the genetic study, the mean (SD) age was 51.4 (10.6) years, 61% were women, and the mean (SD) BMI was 23.7 (3.4) kg/m^2^. The baseline characteristics of the genotyped participants were similar to those of the subset with plasma 25(OH)D concentrations except for prior history of cardiovascular disease, which was an exclusion criterion in the biochemistry study (Table 1). The overall mean plasma 25(OH)D concentration was 62.1 (20.2) nmol/l, consistent with values previously reported in Chinese and in European populations. Mean plasma concentration of 25(OH)D was lower in participants recruited in winter than in summer (57.4 versus 68.4 nmol/l, respectively), but was unrelated to age, sex, physical activity, or percent body fat (S3 Table).

**Table 1:** Selected characteristics for all participants with 25-hydroxyvitamin D (25[OH]D) measured and genetic data in the China Kadoorie Biobank (CKB).

Baseline characteristic	CKB participants with 25(OH)D measured^1^ (*n* = 13,565)	CKB participants with genetic data^2^ (*n* = 82,464)
**Demographic**		
Age, years	53.2 (11.2)	51.4 (10.6)
Women	49.2%	60.5%
Current smoker	47.2%	36.9%
Current drinker	56.8%	53.7%
Body mass index, kg/m^2^	23.6 (3.5)	23.7 (3.4)
Random blood glucose, mmol/l	6.2 (2.8)	6.1 (2.4)
Systolic blood pressure, mm Hg	140.2 (25.9)	131.2 (21.3)
Diastolic blood pressure, mm Hg	82.2 (14.5)	77.8 (11.2)
**Doctor-diagnosed prior disease**		
Heart disease	0.0%	3.0%
Stroke/transient ischemic attack	0.0%	1.8%
Hypertension	15.9%	11.5%
Diabetes	3.7%	3.2%
Cancer	0.0%	0.5%
**Current medication use**		
Statin	0.0%	0.2%
Aspirin	1.3%	1.1%
Blood-pressure-lowering drug	6.1%	4.8%
**Plasma 25(OH)D, nmol/l**	62.0 (20.3)	62.1 (20.2)

Data are given as mean (SD) or percent.

^1^Included in observational analysis of 25(OH)D concentration and diabetes.

^2^Included in Mendelian randomisation (MR) analyses of genetically instrumented 25(OH)D concentration and diabetes; 3,014 individuals with both 25(OH)D measurement and genetic data were included in genetic analyses of 25(OH)D concentrations and MR analyses.

### Association of biochemically measured 25(OH)D concentration with diabetes in CKB

In CKB, biochemically measured plasma 25(OH)D concentration was inversely associated with risk of incident diabetes after adjustment for age, sex, season, area, and additional confounding factors (S4 Fig). The association of 25(OH)D with diabetes was largely unaltered after sequential adjustment for the possible confounders: latitude, sex, SBP, physical activity, age, and percent body fat (S5 Fig). After adjustment for potential confounding factors, a 25-nmol/l higher 25(OH)D concentration was associated with a 9% (95% CI: 0%–18%) lower risk of incident diabetes in CKB, consistent with the 17% (95% CI: 13%–21%) lower risk previously reported in European populations (S4 Table). Combined analysis of both Chinese and European populations suggested that a 25-nmol/l higher biochemically measured plasma 25(OH)D concentration was associated with a 16% (95% CI: 12%–19%) lower risk of diabetes (S4 Table).

### Effect of latitude on 25(OH)D concentration

In CKB, mean plasma 25(OH)D concentration was inversely related to latitude, with about a 2-fold greater mean plasma 25(OH)D concentration in the extreme south area (Haikou) compared with the extreme north area (Harbin) of China (90 nmol/l at 20° versus 49 nmol/l at 46°; S5 Table).

### Effect of genetic variants on 25(OH)D in CKB

*DHCR7*-rs12785878, *CYP2R1*-rs10741657, and *GC/DBP*-rs2282679 were significantly associated with plasma 25(OH)D concentration (Table 2). Although *CYP24A1*-rs6013897 was not significantly associated with 25(OH)D concentration, the direction of effect was consistent with previous studies [14]. The mean (SE) per allele effect on 25(OH)D concentration of the genetic score for the 2 synthesis SNPs was 2.87 (0.39) nmol/l (F-statistic = 21.9), and for the genetic score for all 4 SNPs was 3.54 (0.32) nmol/l (F-statistic = 93.1), in CKB. The estimates were consistent with the previously reported per allele effects on 25(OH)D concentration of these genetic scores in Europeans: 2.72 (0.41) and 3.10 (0.29) nmol/l, respectively [5]. The 25(OH)D-increasing allele frequencies were 0.46 for *DHCR7*-rs12785878, 0.36 for *CYP2R1*-rs10741657, 0.84 for *CYP24A1*-rs6013897, and 0.70 for *GC/DBP*-rs2282679, respectively, in CKB overall, and showed associations towards being higher in those living in northern than in southern latitudes in China (S5 Table). In contrast, the corresponding frequencies for both transport and metabolism SNPs varied little by latitude. There were weak associations towards higher mean effects for the 2-SNP score on 25(OH)D concentrations with more northerly latitude, where the overall concentration of 25(OH)D was higher (although there is likely to be some misclassification of latitude in such large-scale genetic studies).

**Table 2:** Association of 25(OH)D SNPs with plasma 25(OH)D concentrations and with cardiometabolic risk factors.

SNP	25(OH)D, nmol/l	SBP, mm Hg	DBP, mm Hg	BMI, kg/m^2^	Waist—hip ratio	Body fat percent	Random blood glucose, mmol/l
**rs12785878 (*DHCR7*)**							
Per T allele	2.84 (0.41)	0.10 (0.10)	0.05 (0.05)	−0.01 (0.02)	−0.00 (0.08)	−0.00 (0.03)	0.00 (0.01)
*p*-Value for trend	2.3 × 10^−12^	0.31	0.32	0.70	0.98	0.99	0.94
**rs10741657 (*CYP2R1*)**							
Per A allele	0.95 (0.42)	−0.02 (0.10)	0.01 (0.06)	0.02 (0.02)	−0.10 (0.08)	0.07 (0.03)	−0.01 (0.01)
*p*-Value for trend	0.03	0.83	0.88	0.21	0.21	0.02	0.38
**rs6013897 (*CYP24A1*)**							
Per A allele	0.51 (0.56)	0.14 (0.13)	0.09 (0.07)	0.00 (0.02)	0.03 (0.11)	−0.01 (0.04)	0.01 (0.01)
*p*-Value for trend	0.37	0.28	0.23	0.89	0.76	0.73	0.37
**rs2282679 (*GC/DBP*)**							
Per T allele	3.59 (0.44)	−0.06 (0.10)	−0.06 (0.06)	0.01 (0.02)	0.11 (0.09)	0.00 (0.03)	0.02 (0.01)
*p*-Value for trend	2.2 × 10^−16^	0.58	0.28	0.39	0.20	0.99	0.09

Data are given as mean (SE) and *p*-value. For SNP rs12785878: *n* = 3,004 for 25(OH)D, *n* = 82,448 for SBP, DBP, BMI, and waist—hip ratio, *n* = 82,393 for body fat percent, and *n* = 81,637 for random blood glucose. For SNP rs10741657: *n* = 3,011 for 25(OH)D, *n* = 82,452 for SBP, DBP, BMI, and waist—hip ratio, *n* = 82,397 for body fat percent, and *n* = 81,641 for random blood glucose. For SNP rs6013897: *n* = 3,014 for 25(OH)D, *n* = 82,396 for SBP, DBP, BMI, and waist—hip ratio, *n* = 82,341 for body fat percent, and *n* = 81,585 for random blood glucose. For SNP rs2282679: *n* = 3,013 for 25(OH)D, *n* = 82,296 for SBP, DBP, BMI, and waist—hip ratio, *n* = 82,242 for body fat percent, and *n* = 81,485 for random blood glucose. All values are adjusted for age, sex, and season, and stratified by area.

25(OH)D, 25-hydroxyvitamin D; DBP, diastolic blood pressure; SBP, systolic blood pressure.

None of the 4 SNPs was associated with differences in blood pressure, BMI, WHR, percent body fat, or random plasma glucose concentration in CKB (Table 2).

### Genetic variants and risk of diabetes in CKB

In CKB, none of the individual variants were significantly associated with risk of diabetes (*n* = 5,566), with per allele adjusted ORs (95% CIs) of 0.97 (0.93–1.01) for *DHCR7*-rs12785878, 1.00 (0.96–1.04) for *CYP2R1*-rs10741657, 1.00 (0.95–1.05) for *CYP24A1*-rs6013897, and 1.01 (0.96–1.05) for *GC/DBP*-rs2282679 (S6 Fig). The per allele adjusted OR (95% CI) of the genetic score for the 2 synthesis SNPs was 0.97 (0.93–1.01) and for the genetic score for all 4 SNPs was 0.99 (0.96–1.02).

### Genetic variants and risk of diabetes in an updated meta-analysis of all studies

**Fig 1:**
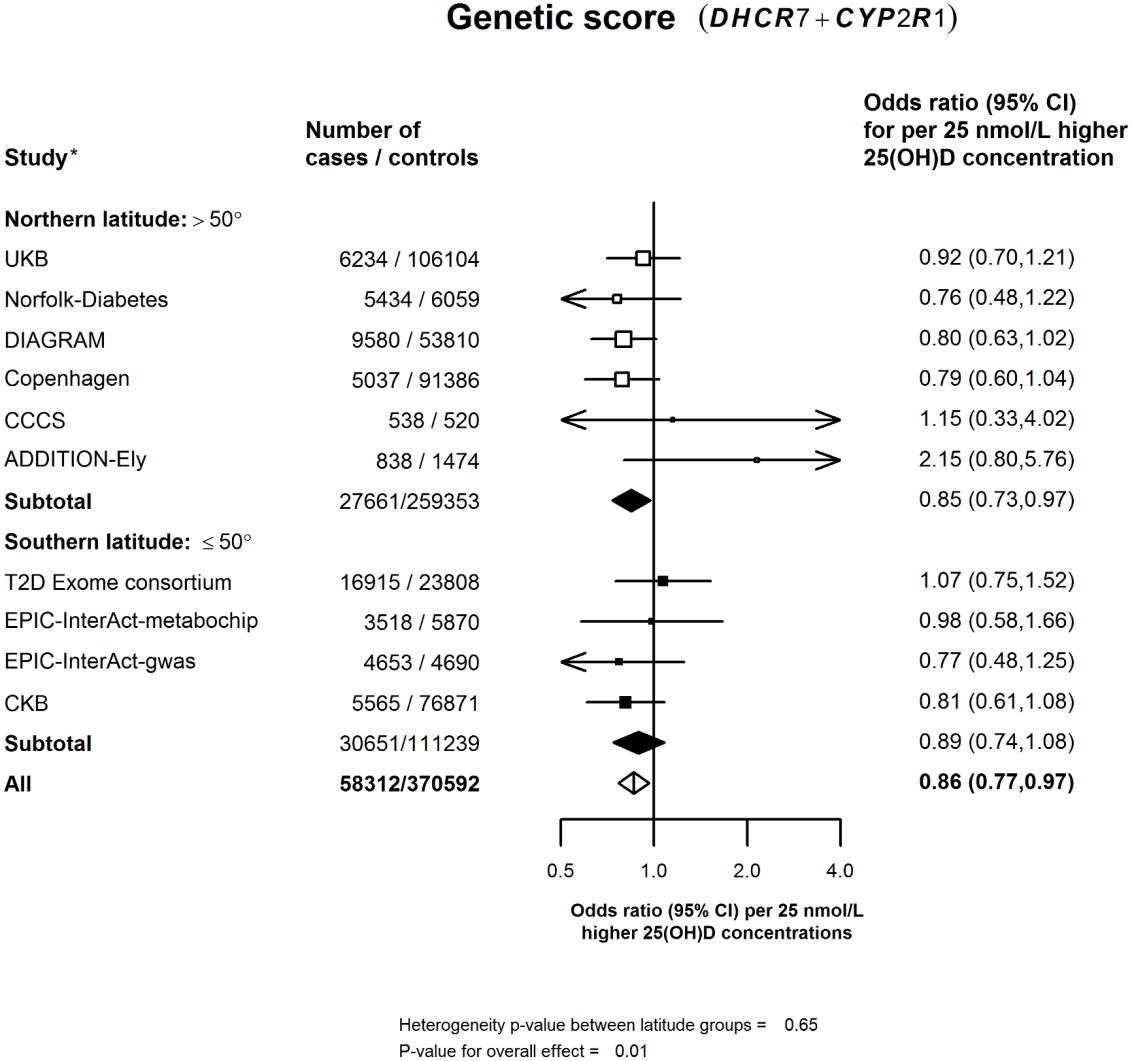
Association of genetic score using synthesis SNPs for 25(OH)D concentration with risk of diabetes in a meta-analysis of all studies per 25-nmol/l higher genetically instrumented 25(OH)D concentration. Values shown are the odds ratios (95% CIs) per 25-nmol/l higher 25(OH)D concentration among studies stratified by latitude into northern (>50°) or southern latitude (≤50°). The area of the squares is proportional to the inverse variance of each effect size. *The effects of all SNPs on risk of diabetes in Chinese and European populations were weighted by their effects on 25(OH)D concentration. 25(OH)D, 25-hydroxyvitamin D; CCCS, Cambridgeshire case—control study; CKB, China Kadoorie Biobank; DIAGRAM, Diabetes Genetics Replication and Meta-analysis; UKB, UK Biobank.

**Fig 2:**
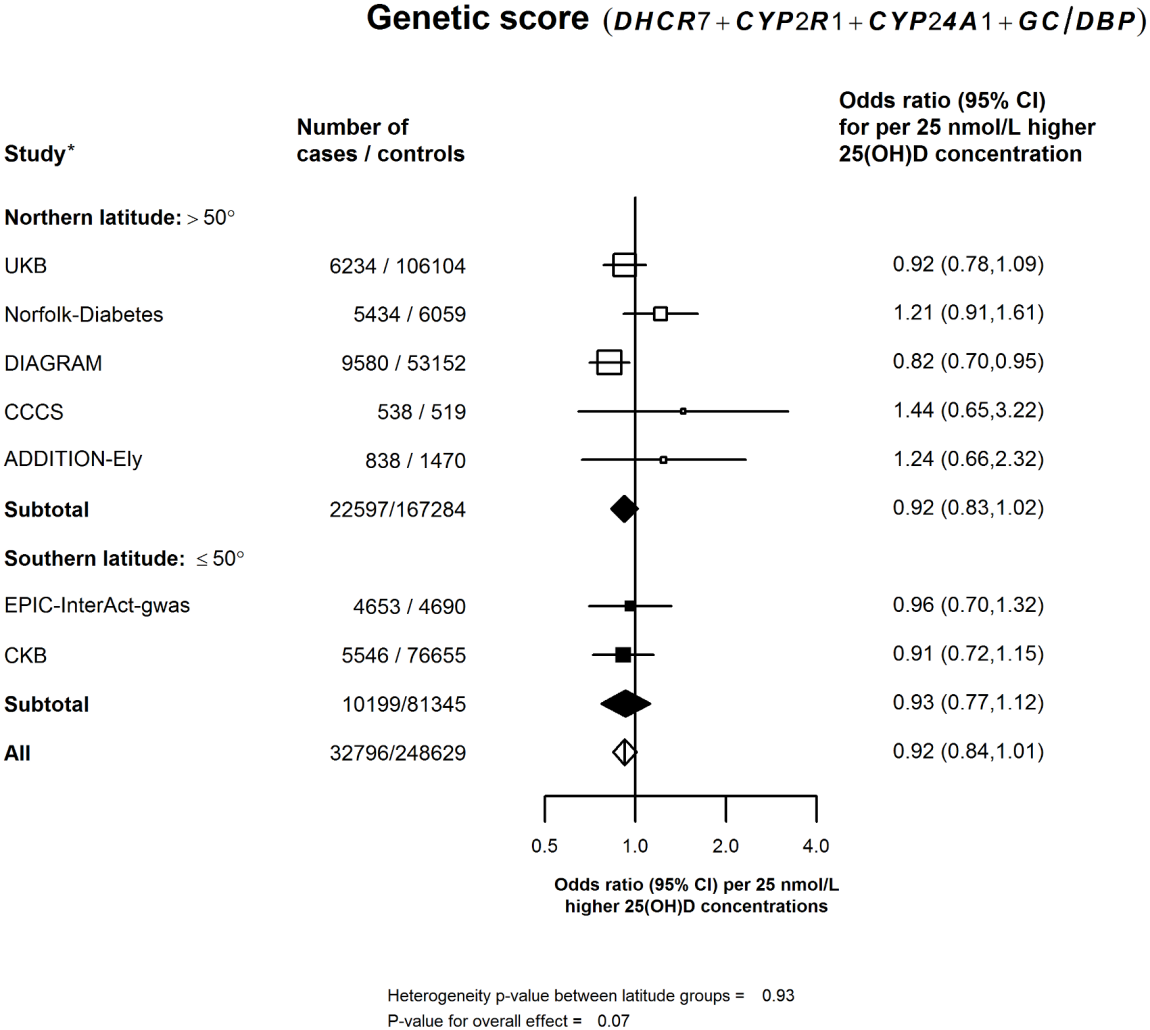
Association of genetic score using all 4 SNPs for 25(OH)D concentration with risk of diabetes in a meta-analysis of all studies per 25-nmol/l higher genetically instrumented 25(OH)D concentration. Values shown are the odds ratios (95% CIs) per 25-nmol/l higher 25(OH)D concentration among studies stratified by latitude into northern (>50°) or southern latitude (≤50°). Symbols and conventions as in Fig 1. *The effects of all SNPs on risk of diabetes in Chinese and European populations were weighted by their effects on 25(OH)D concentration. 25(OH)D, 25-hydroxyvitamin D; CCCS, Cambridgeshire case—control study; CKB, China Kadoorie Biobank; DIAGRAM, Diabetes Genetics Replication and Meta-analysis; UKB, UK Biobank.

**Fig 3:**
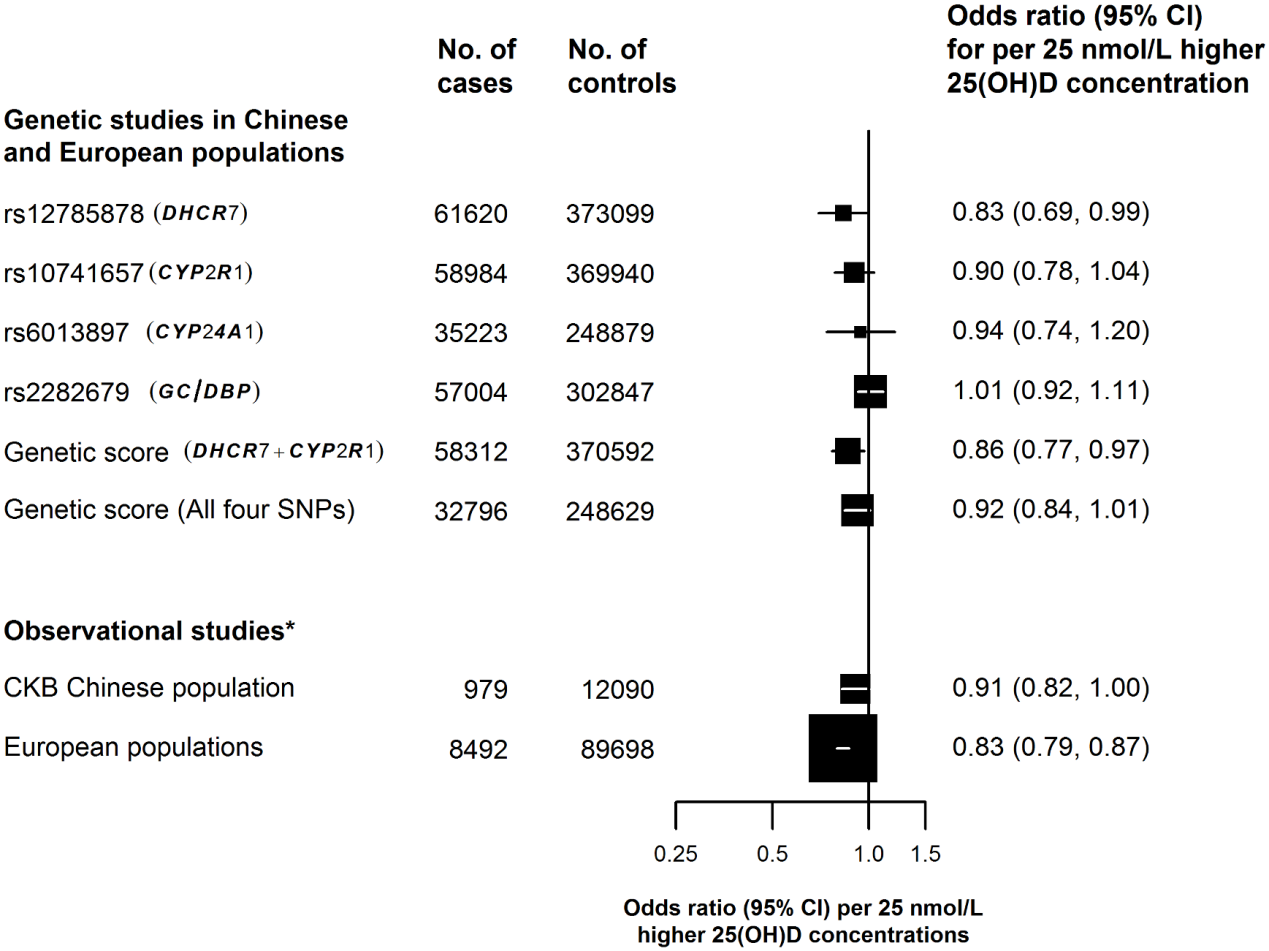
Comparison of the associations of biochemically measured and genetically instrumented 25-nmol/l higher plasma 25(OH)D concentrations with risk of diabetes. *The full details of the adjustments in the observational analyses are provided in S3 Table. Other symbols and conventions as in Fig 1. 25(OH)D, 25-hydroxyvitamin D; CKB, China Kadoorie Biobank.

In the updated meta-analysis of all 10 studies, the primary analysis involved a total of 58,312 cases and 370,592 controls (S2 Fig), and a 25-nmol/l higher genetically instrumented plasma 25(OH)D concentration using the 2 synthesis SNPs was associated with a 14% (95% CI: 3%–23%) lower risk of diabetes (*p* = 0.01; Fig 1). The secondary analyses, involving a meta-analysis of 7 of the 10 studies, including 32,796 cases and 248,629 controls with complete data on all synthesis, transport, and catabolism SNPs, indicated that an equivalent difference in 25(OH)D was associated with only an 8% (95% CI: −1% to 16%) lower risk of diabetes (*p* = 0.07) (Fig 2). Analyses based on the 3 SNPs available in all 10 studies gave a similar result (S7 Fig). The estimates for the 2- versus 4-SNP genetic scores in the original and updated meta-analyses were concordant with each other and with the results from the observational studies for an equivalent difference in plasma 25(OH)D concentration in both Chinese and in European populations (Fig 3).

### Sensitivity analyses

S8 Fig shows some evidence of pleiotropy for the transport and catabolism SNPs in Chinese and European populations, and the *p*-values for the MR—Egger intercept (a test of directional pleiotropy) were 0.064 and 0.045 for the 4-SNP and 3-SNP genetic scores, respectively, suggesting possible pleiotropy for the transport and catabolism SNPs.

## Discussion

This MR study of genetic variants influencing the synthesis of plasma 25(OH)D concentration, involving over 58,000 cases and 370,000 controls, demonstrated concordant risks of diabetes for genetically instrumented differences in 25(OH)D using synthesis SNPs and biochemically measured differences, and provides novel evidence for a causal protective effect of higher 25(OH)D concentrations on risk of diabetes. The present meta-analysis included substantially more cases than a previously reported meta-analysis (involving only 28,144 cases and 76,344 controls) [5] and reported that a 25-nmol/l higher genetically instrumented plasma 25(OH)D concentration using the synthesis SNPs was associated with a 14% (95% CI: 3%–23%) lower risk of diabetes. The concordance of genetic studies of 25(OH)D-raising alleles using the synthesis SNPs and risk of diabetes in both Chinese and European populations indicates a protective effect of higher plasma 25(OH)D concentrations for risk of diabetes.

The results of the present study indicate that a genetically instrumented 25-nmol/l higher plasma 25(OH)D concentration using all 4 SNPs was associated with only an 8% (95% CI: −1% to 16%) lower risk of diabetes (based on half the number of cases). However, several studies have reported that both the transport and catabolism SNPs show biological evidence of pleiotropy [13,16,17]. Analysis of the associations of the transport and catabolism SNPs with diabetes also shows modest statistical evidence of pleiotropy (S8 Fig). Previous studies have suggested biological evidence of pleiotropy for *GC/DBP*-rs2282679, which influences vitamin D binding protein, resulting in discrepant ratios of free to total plasma 25(OH)D concentrations and alteration in feedback control of 25(OH)D concentrations [13,16,17]. In addition, vitamin D binding protein also carries actin (a chemotactic factor implicated in inflammation), which may affect diabetes independent of the effect on 25(OH)D concentrations [28,29].

Individuals living near the equator, and who are also routinely exposed to sunlight, have substantially higher plasma 25(OH)D concentrations than those living in northern latitudes (with those living above about 35° having little or no vitamin D production from skin during the winter months) [2]. In addition to showing an almost 2-fold difference in mean plasma 25(OH)D concentration (90 nmol/l at 20° versus 49 nmol/l at 46°), the present analysis of 25(OH)D concentrations in CKB demonstrated that latitude influenced the 25(OH)D-increasing allele frequencies, indicating a possible selection effect. However, the present study had limited power to detect an effect of latitude on the association of the 2-SNP genetic score for 25(OH)D with risk of diabetes either in CKB or in the meta-analysis of all studies. It is possible that incidence of type 2 diabetes may have been underestimated in this MR study in the absence of population-wide screening programmes for diabetes in the populations studied.

While a previous study reported modest associations of a 2-SNP genetic score with blood pressure and risk of hypertension [30], the present study demonstrated no effect of the individual variants on blood pressure, blood glucose, or any measure of adiposity in the Chinese population. None of these variants were associated with fasting blood glucose or insulin concentrations in previously published studies in Western populations [23], but many variants affecting risk of diabetes have no detectable effect on fasting glucose or insulin concentrations in Western populations, so this should not be regarded as an indication of inconsistency [22]. Meta-analyses of genetic studies conducted in different populations cannot exclude the possibility of uncorrected population stratification in individual studies. However, the analyses of the individual variants and both the 2-SNP and 4-SNP genetic scores in CKB were conducted within areas, and area-specific estimates were combined using inverse-variance weighting, which should minimise effects of population stratification for the CKB analyses.

The association between vitamin D and diabetes is biologically plausible [31,32]. In vitro studies showed that treatment with 1,25(OH)_2_D, the bioactive form of vitamin D, can stimulate insulin gene expression and regulate calcium flux in β cells in human pancreatic cell lines, which is important for insulin production and secretion [32,33]. Likewise, vitamin D—deficient rats had improved insulin status after injection with 1,25-dihydroxyvitamin D3 [33]. Hence, improving vitamin D status might be a potential method to improve β cell function and subsequently decrease the risk of diabetes.

The findings of the present study have potentially important implications for public health policies on food fortification with vitamin D for prevention of diabetes (in addition to effects on bone health and other disease outcomes). Population-wide supplementation with vitamin D could afford a simple and cost-effective approach for prevention of diabetes in vitamin D—deficient populations. Indeed, some studies have already suggested that the introduction of vitamin D fortification in Finland, a population with limited exposure to sunlight, attenuated secular trends of increasing incidence of type 1 diabetes [34].

Previous randomised trials of vitamin D supplementation were not designed to assess the effects on risk of incident diabetes [8–12]. However, some large trials have tested effects of relatively low doses of vitamin D on diabetes or on glycaemic traits [8–12]. However, the doses of vitamin D used in several of these trials, typically raised 25(OH)D by only 10–20 nmol/l and, hence, may not have been sufficient to produce any detectable effects on the incidence of diabetes or on glycaemic traits [35]. Moreover, the duration of these trials may not have been long enough to detect effects on diabetes compared to the lifelong differences in 25(OH)D concentrations in MR studies. The results of ongoing or planned large-scale trials testing the effects of higher doses of vitamin D (involving at least 2,000 IU daily) are needed to address the effect of vitamin D on incidence of diabetes. The D2d trial in the US is currently assessing whether supplementation with 4,000 IU vitamin D or placebo delays the onset of type 2 diabetes in 2,382 individuals with pre-diabetes [36]. Ongoing trials of vitamin D supplementation may not be able to accrue sufficient numbers of type 2 diabetes cases for reliable assessment of the effect of vitamin D on the risk of type 2 diabetes, and additional trials with larger numbers of such cases may be needed. The results of meta-analysis of ongoing and future trials of vitamin D supplementation are required before advocating use of vitamin D supplements (or food fortification) for the prevention of diabetes.

## Supporting information

S1 Fig Schematic representation of the function of genetic variants for proteins that influence circulating 25(OH)D concentrations. (DOCX)

S2 Fig Flow diagram of included studies. (DOCX)

S3 Fig Details of participating studies in Chinese and European populations with plasma 25(OH)D concentrations and 2 synthesis SNPs for 25(OH)D and subset with 4 SNPs for 25(OH)D and risk of diabetes. (DOCX)

S4 Fig Association of tertiles of biochemically measured 25(OH)D concentration with incidence of diabetes after adjustment for confounding factors in the China Kadoorie Biobank. The odds ratios are adjusted for age, sex, latitude, season, SBP, physical activity, and body fat percentage. The dotted line is from a weighted linear regression with weights based on the inverse variance of the estimate (*p*-value for non-linearity = 0.11). The odds ratios are presented using floated variances, which does not change the point estimates but assigns a standard error to the reference category. The numbers above the vertical lines are point estimates for odds ratios, and the numbers below the lines are numbers of events.
(PDF)

S5 Fig Impact of sequential adjustment for confounding factors on the observational association of biochemically measured 25(OH)D concentration with diabetes in the China Kadoorie Biobank. Symbols and conventions as in Fig 1. The covariates were entered into a linear regression model in a forward stepwise manner (with entry criterion of *p* ≤ 0.05). The covariates included in the stepwise regression were as follows: age as a continuous variable and in 5-year age groups, sex, season, systolic blood pressure, smoking, body mass index, body fat percent, latitude, and total physical activity. This analysis was based on plasma 25(OH)D concentrations from 13,565 individuals.
(PDF)

S6 Fig Association of individual variants and of genetic scores for plasma 25(OH)D concentration with risk of diabetes in the China Kadoorie Biobank. Symbols and conventions as in Fig 1. The genetic scores were weighted by the individual per allele effects of rs12785878, rs10741657, rs6013897, and rs2282679 on 25(OH)D concentration in CKB.
(PDF)

S7 Fig Association of genetic score using 2 synthesis SNPs and the lead transport SNP for 25(OH)D concentration with risk of diabetes in a meta-analysis of all available studies per 25-nmol/l higher genetically instrumented 25(OH)D concentration. Symbols and conventions as in Fig 1. *Both CKB and all other studies are weighted by the effects of each SNP on 25(OH)D concentration.
(PDF)

S8 Fig Scatterplot of the associations of the per allele effects of the 2 synthesis SNPs and the transport and catabolism SNPs for 25(OH)D concentration with risk of diabetes in European and Chinese populations, by their effect on 25(OH)D concentration. Symbols for synthesis SNPs are shown in black, and those for transport and catabolism SNPs are shown in red. Open symbols are used for the Chinese population, and closed symbols are used for the European population. Different shape symbols are used for each SNP.
(PDF)

S1 Table PRISMA checklist. (DOC)

S2 Table Studies included in the meta-analysis of the association of the 2-SNP genetic score influencing plasma 25(OH)D concentration with risk of diabetes. (DOCX)

S3 Table Plasma 25(OH)D concentration by relevant confounders in CKB. (DOCX)

S4 Table Comparison of observational association of plasma 25(OH)D concentration with risk of diabetes in the CKB study with the meta-analysis of prospective European cohorts. (DOCX)

S5 Table Allele frequency of individual SNPs and effect of genetic scores on mean plasma 25(OH)D concentration using the synthesis SNPs and all four 25(OH)D SNPs, by latitude in CKB. (DOCX)

## Abbreviations

25(OH)D: 25-hydroxyvitamin D
CKB: China Kadoorie Biobank
DBP: diastolic blood pressure
MR: Mendelian randomisation
OR: odds ratio
SBP: systolic blood pressure
SNP: single nucleotide polymorphism
WHR: waist—hip ratio
