# Supplementary material for: Association of vitamin D with risk of type 2 diabetes: A Mendelian randomisation study in European and Chinese adults
Source: PLoS Med. 2018 May 2;15(5):e1002566. doi: 10.1371/journal.pmed.1002566 (PMC5931494; doi:10.1371/journal.pmed.1002566)
Supplement: S1 Fig — (DOCX) [file pmed.1002566.s001.docx]

**S1 Fig: Schematic representation of the function of genetic variants for proteins that influence circulating 25(OH)D concentrations**

**Synthesis**

UVB

7-dehydroxycholesterol

Pre-vitamin D3

Vitamin D3

7-dehydrocholesterol reductase (***DHCR7***)

Skin

Diet

Liver

25-hydroxylase

(***CYP2R1***)

Vitamin D

Circulation

**Transport/catabolism**

Transport

Vitamin D binding protein

(***GC/DBP***)

25(OH)D

Kidneys

24-hydroxylase

(***CYP24A1***)

Catabolism

Clearance

Activation

Calcitroic

acid

1,25(OH)_2_D3

(Calcitriol)

| **Effect on 25(OH)D concentrations** | **Synthesis** | | **Catabolism/Transport** | |
| --- | --- | --- | --- | --- |
|  | ***DHCR7*** | ***CYP2R1*** | ***CYP24A1*** | ***GC/DBP*** |
| Enzyme | 7-dehydrocholesterol reductase | 25-hydroxylase | 24-hydroxylase | Vitamin D binding protein |
| SNP | rs12785878 | rs10741657 | rs6013897 | rs2282679 |
| Effect Allele | T | A | A | T |
| Biochemical function | Reduced synthesis of pre-vitamin D3 | Reduced synthesis of 25(OH)D | Reduced clearance of 25(OH)D | Reduced binding of 25(OH)D |
| Possible pleiotropy |  |  | Altered feedback involving phosphate/FGF-23 that influences proportions of “free”/”total” vitamin D | Altered carriage of “free” vitamin D and carriage of pro-inflammatory proteins |
