# Supplementary material for: Association of vitamin D with risk of type 2 diabetes: A Mendelian randomisation study in European and Chinese adults
Source: PLoS Med. 2018 May 2;15(5):e1002566. doi: 10.1371/journal.pmed.1002566 (PMC5931494; doi:10.1371/journal.pmed.1002566)
Supplement: S2 Fig — (DOCX) [file pmed.1002566.s002.docx]

### S2 Fig: Flow diagram of included studies

Articles or meeting abstracts retrieved through searching on PubMed and Web of Science (n = 282)

Title and abstract screened

Excluded after title and abstract screening (n = 277)

- Reviews, editorials, and animal or in vitro study (n =114)
- Irrelevant exposures or outcomes (n = 163)

Full articles or meeting abstracts obtained and reviewed (n = 5)

- Tromsø study
- EPIC-German study
- EPIC-Norfolk study
- Copenhagen study
- A meta-analysis of 6 European studies

Summary data from CKB, T2D Exome consortium and UK- Biobank study

Excluded after full articles review (n = 3)

- Per-allele effect size or genotype distribution
  in cases and controls for CYP2R1-
  rs10741657 and DHCR7-rs12785878 not available (n = 1)
- Study participants overlap (n=2)

Studies included in the current meta-analysis (n = 10)

- - A meta-analysis of 6 European studies
  - Copenhagen study
  - T2D Exome consortium
  - UK-Biobank study
  - CKB
