# Supplementary material for: Association of vitamin D with risk of type 2 diabetes: A Mendelian randomisation study in European and Chinese adults
Source: PLoS Med. 2018 May 2;15(5):e1002566. doi: 10.1371/journal.pmed.1002566 (PMC5931494; doi:10.1371/journal.pmed.1002566)
Supplement: S3 Fig — (DOCX) [file pmed.1002566.s003.docx]

**S3 Fig: Details of participating studies in Chinese and European populations with plasma 25(OH)D concentrations and 2 synthesis SNPs for 25(OH)D levels and subset with 4 SNPs for 25(OH)D and risk of diabetes**

Meta-analysis of 2 synthesis SNPs for 25(OH)D and diabetes in Chinese and European populations

Cases / controls: 58,312 / 370,592

Meta-analysis of 4 SNPs for 25(OH)D and diabetes in Chinese and European populations

Cases / controls: 32,796 / 248,629

Genetic studies (n=6)
of 4 SNPs
for 25(OH)D and
risk of diabetes

Total population 13,565

Diabetes cases 979

Controls 12,090

Observational
studies of 25(OH)D
and risk of diabetes

Genetic studies of
SNPs for 25(OH)D
and risk of diabetes

Chinese population (CKB)

n=512,891

UKB 6234 / 106,104

Norfolk Diabetes 5434 / 6059

DIAGRAM 9580 / 53,810

Copenhagen 5037 / 91,386

CCCS 538 / 520

ADDITION-Ely 838 / 1474

T2D Exome Consortium 16,915 / 23,808

EPIC-InterAct-metabochip 3518 / 5870

EPIC-InterAct-gwas 4653 / 4690

UKB 6234 / 106,104

Norfolk Diabetes 5434 / 6059

DIAGRAM 9580 / 53,152

-

CCCS 538 / 519

ADDITION-Ely 838 / 1470

-

-

EPIC-InterAct-gwas 4653 / 4690

Genetic studies (n=9)
of 2 synthesis SNPs
for 25(OH)D and
risk of diabetes

European populations

9 studies

Total population 82,404

Diabetes cases 5565

Controls 76,871
