# Supplementary material for: Association of vitamin D with risk of type 2 diabetes: A Mendelian randomisation study in European and Chinese adults
Source: PLoS Med. 2018 May 2;15(5):e1002566. doi: 10.1371/journal.pmed.1002566 (PMC5931494; doi:10.1371/journal.pmed.1002566)
Supplement: S4 Fig — The odds ratios are adjusted for age, sex, latitude, season, SBP, physical activity, and body fat percentage. The dotted line is from a weighted linear regression with weights based on the inverse variance of the estimate (p-value for non-linearity = 0.11). The odds ratios are presented using floated variances, which does not change the point estimates but assigns a standard error to the reference category. The numbers above the vertical lines are point estimates for odds ratios, and the numbers below the lines are numbers of events. (PDF) [file pmed.1002566.s004.pdf]

**S4 Fig: Association of tertiles of biochemically measured 25(OH)D concentration with incidence of diabetes after adjustment for confounding factors in the China Kadoorie Biobank**

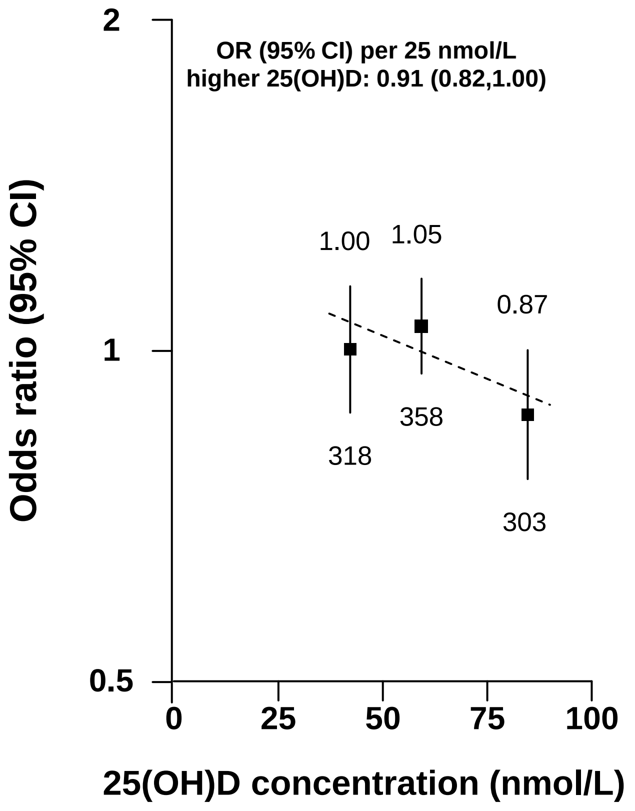


The odds ratios are adjusted for age, sex, latitude, season, SBP, physical activity, and body fat percentage. Dotted line is from a weighted linear regression with weights based on the inverse variance of the estimate; P-value for non−linearity = 0.11. The odds ratios are presented using floated variances which does not change the point estimates, but assigns a standard error to the reference category. The numbers above the vertical lines are point estimates for odds ratios, and the numbers below the lines are numbers of events.
