# Supplementary material for: Association of vitamin D with risk of type 2 diabetes: A Mendelian randomisation study in European and Chinese adults
Source: PLoS Med. 2018 May 2;15(5):e1002566. doi: 10.1371/journal.pmed.1002566 (PMC5931494; doi:10.1371/journal.pmed.1002566)
Supplement: S5 Fig — Symbols and conventions as in Fig 1. The covariates were entered into a linear regression model in a forward stepwise manner (with entry criterion of p ≤ 0.05). The covariates included in the stepwise regression were as follows: age as a continuous variable and in 5-year age groups, sex, season, systolic blood pressure, smoking, body mass index, body fat percent, latitude, and total physical activity. This analysis was based on plasma 25(OH)D concentrations from 13,565 individuals. (PDF) [file pmed.1002566.s005.pdf]

S5 Fig: Impact of sequential adjustment for confounding factors on the observational association of biochemically measured 25(OH)D concentration with diabetes in the China Kadoorie Biobank

Incident diabetes
(979 cases; 12,090 controls)

**Adjustment for confounding factors**	**Odds ratio (95% CI) for per 25 nmol/L higher 25(OH)D concentration**	χ^2_1
Unadjusted	0.93 (0.86,1.01)	2.6
+ latitude	0.83 (0.76,0.91)	15.4
+ sex	0.86 (0.78,0.95)	9.1
+ systolic blood pressure	0.89 (0.81,0.98)	5.6
+ physical activity	0.88 (0.80,0.97)	7.0
+ age	0.88 (0.80,0.97)	6.7
+ fat percent	0.91 (0.82,1.00)	3.5

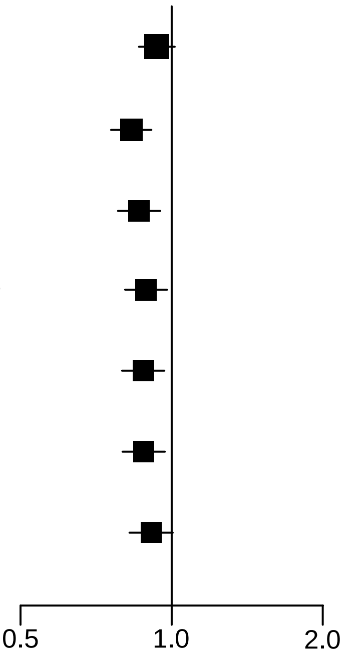

Odds ratio (95% CI) per 25 nmol/ L higher 25(OH)D concentration

The covariates were entered into a linear regression model in a forward stepwise manner (with entry criterion of p≤0.05). The covariates included in the stepwise regression were as follows: age as a continuous variable and in 5−year age groups, sex, season, systolic blood pressure, smoking, body mass index, body fat percent, latitude, and total physical activity. This analysis was based on plasma 25(OH)D concentrations from 13,565 individuals.
