# Supplementary material for: Association of vitamin D with risk of type 2 diabetes: A Mendelian randomisation study in European and Chinese adults
Source: PLoS Med. 2018 May 2;15(5):e1002566. doi: 10.1371/journal.pmed.1002566 (PMC5931494; doi:10.1371/journal.pmed.1002566)
Supplement: S6 Fig — Symbols and conventions as in Fig 1. The genetic scores were weighted by the individual per allele effects of rs12785878, rs10741657, rs6013897, and rs2282679 on 25(OH)D concentration in CKB. (PDF) [file pmed.1002566.s006.pdf]

S6 Fig: Association of individual genetic variants and of genetic scores for plasma 25(OH)D concentration with risk of diabetes in the China Kadoorie Biobank

Variant or genetic score	Number of cases / controls	Odds ratio (95% CI) per allele	Allele (freq) in CKB**	Mean (SE) 25(OH)D in CKB
**rs12785878** (*DHCR7*)	5566 / 76,882	0.97 (0.93,1.01)	T (0.46)	2.8 (0.41)
**rs10741657** (*CYP2R1*)	5565 / 76,887	1.00 (0.96,1.04)	A (0.36)	0.9 (0.42)
**rs6013897** (*CYP24A1*)	5560 / 76,836	1.00 (0.95,1.05)	T (0.84)	0.5 (0.56)
**rs2282679** (*GC/DBP*)	5553 / 76,743	1.01 (0.96,1.05)	T (0.70)	3.6 (0.44)
**Weighted genetic scores***				
DHCR7+CYP2R1	5565 / 76,871	0.97 (0.93,1.01)	–	2.9 (0.39)
DHCR7+CYP2R1+CYP24A1+GC/DBP	5546 / 76,655	0.99 (0.96,1.02)	–	3.5 (0.32)

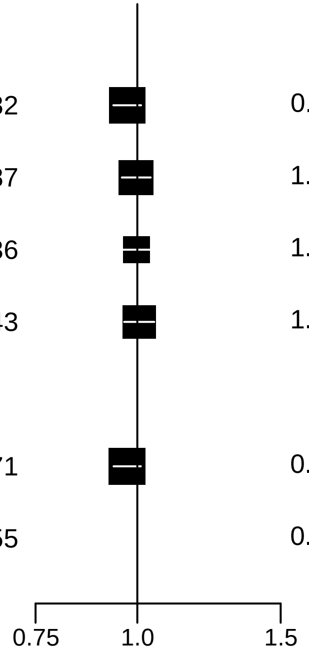

*The genetic scores were weighted by the individual per allele effects of rs12785878, rs10741657, rs6013897 and rs2282679 on 25(OH)D concentration in CKB. The rs10741657 had 12 missing genotypes, rs12785878 had 16 missing genotypes, rs6013897 had 68 missing genotypes, and rs2282679 had 168 missing genotypes.

**25(OH)D increasing allele
