# Supplementary material for: Association of vitamin D with risk of type 2 diabetes: A Mendelian randomisation study in European and Chinese adults
Source: PLoS Med. 2018 May 2;15(5):e1002566. doi: 10.1371/journal.pmed.1002566 (PMC5931494; doi:10.1371/journal.pmed.1002566)
Supplement: S7 Fig — Symbols and conventions as in Fig 1. *Both CKB and all other studies are weighted by the effects of each SNP on 25(OH)D concentration. (PDF) [file pmed.1002566.s007.pdf]

S7 Fig: Association of genetic score using two synthesis SNPs and the lead transport SNP for plasma 25(OH)D concentration with risk of diabetes in a meta-analysis of all studies per 25 nmol/L higher genetically instrumented 25(OH)D concentration

Genetic score $(DHCR7 + GC/DBP + CYP2R1)$

Study	Number of cases / controls	Odds ratio (95% CI) for per 25 nmol/L higher 25(OH)D concentration
**Northern latitude:** $> 50°$		
UKB	6234 / 106,104	0.96 (0.81,1.14)
Norfolk−Diabetes	5434 / 6059	1.04 (0.77,1.40)
DIAGRAM	9580 / 53,152	0.86 (0.73,1.01)
Copenhagen	3633 / 23,626	0.77 (0.60,1.00)
CCCS	511 / 499	1.16 (0.50,2.68)
ADDITION−Ely	838 / 1470	1.59 (0.83,3.07)
**Subtotal**	26230/190,910	0.91 (0.83,1.01)
**Southern latitude:** $\leq 50°$		
T2D Exome consortium	16915 / 23,808	1.10 (0.88,1.39)
EPIC−InterAct−metabochip	3514 / 5867	0.92 (0.63,1.32)
EPIC−InterAct−gwas	4653 / 4690	0.88 (0.63,1.22)
CKB*	5552 / 76,716	0.91 (0.72,1.15)
**Subtotal**	30634/111,081	0.97 (0.84,1.11)
**All**	**56864/301,991**	**0.93 (0.86,1.01)**

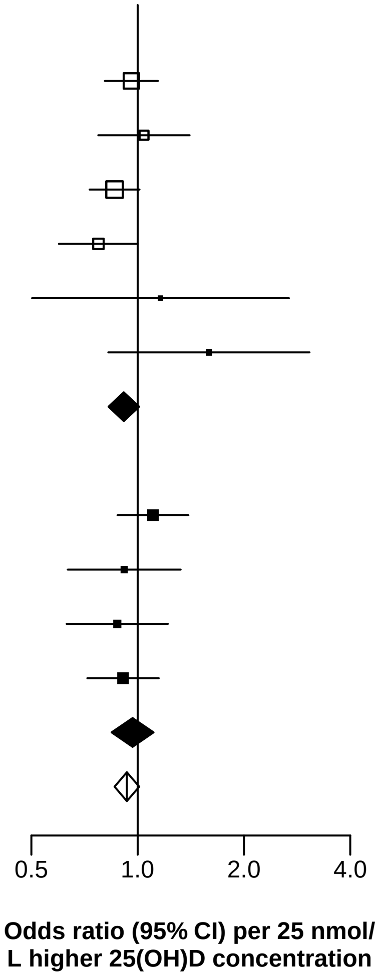

Heterogeneity p-value between latitude groups = 0.51
P−value for overall effect = 0.09

*Both CKB and all other studies are weighted by the effects of each SNP on 25(OH)D concentration.
