# Supplementary material for: Association of vitamin D with risk of type 2 diabetes: A Mendelian randomisation study in European and Chinese adults
Source: PLoS Med. 2018 May 2;15(5):e1002566. doi: 10.1371/journal.pmed.1002566 (PMC5931494; doi:10.1371/journal.pmed.1002566)
Supplement: S8 Fig — Symbols for synthesis SNPs are shown in black, and those for transport and catabolism SNPs are shown in red. Open symbols are used for the Chinese population, and closed symbols are used for the European population. Different shape symbols are used for each SNP. (PDF) [file pmed.1002566.s008.pdf]

S8 Fig: Scatterplot of the associations of the per allele effects of the two synthesis SNPs and the transport and catabolism SNPs for 25(OH)D concentration with risk of diabetes in European and Chinese populations, by their effect on 25(OH)D concentration

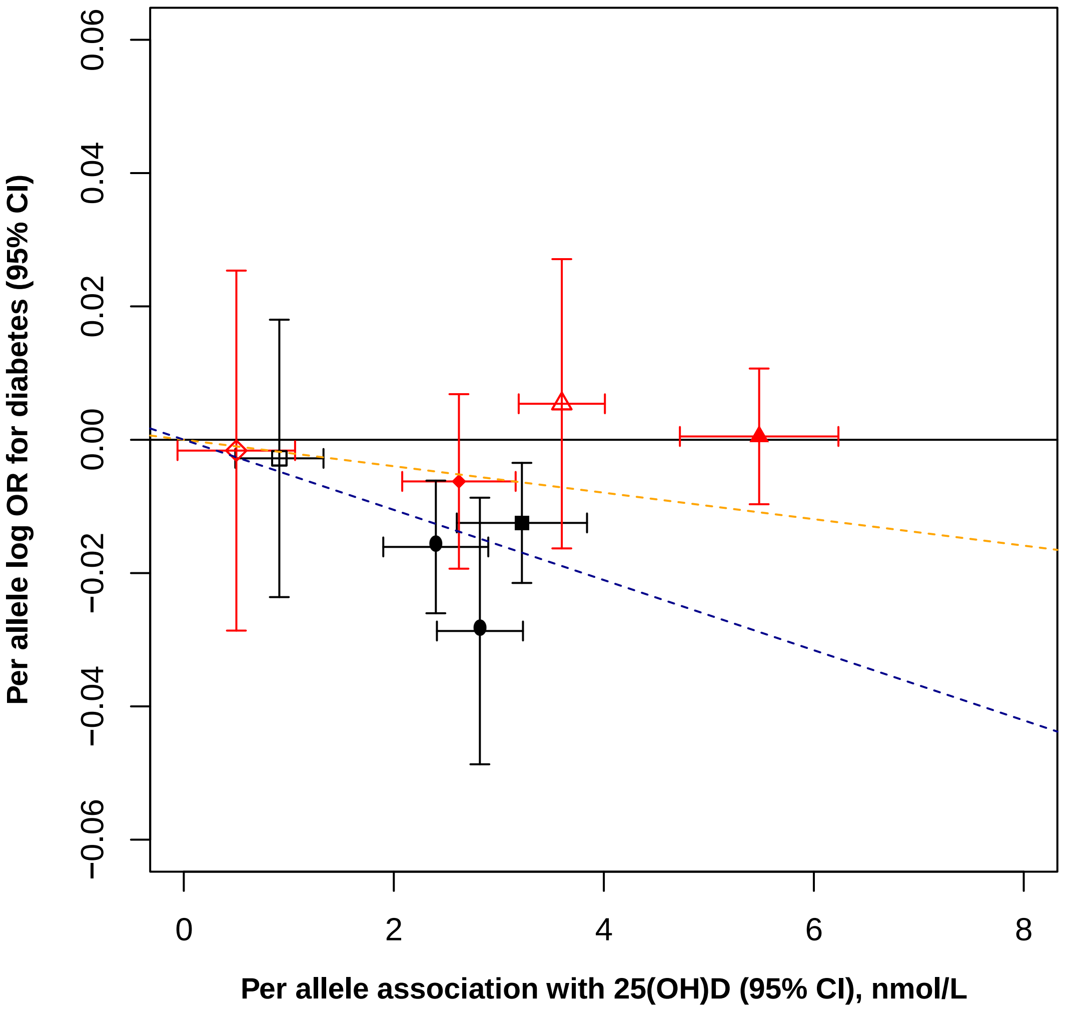

Square: CYP2R1−rs10741657; Diamond: CYP24A1−rs6013897; Circle: DHCR7−rs12785878; Triangle: GC/DBP−rs2282679

Red: Transport/Catabolism SNPs; Black: Synthesis SNPs; Closed symbols: Europeans; Open symbols: Chinese

Ten studies had information for DHCR7, CYP2R1 (synthesis SNPs), 10 studies had information for GC/DBP and 7 studies for CYP24A1 (transport/catabolism SNPs)

Orange dashed line: The slope from an inverse-variance weighted linear regression of per allele log OR for diabetes for all 4 SNPs on 25(OH)D concentrations with the line forced through the origin

Blue dashed line: The slope from an inverse-variance weighted linear regression of per allele log OR for diabetes for synthesis SNPs only on 25(OH)D concentrations with the line forced through the origin
