# Supplementary material for: Association of vitamin D with risk of type 2 diabetes: A Mendelian randomisation study in European and Chinese adults
Source: PLoS Med. 2018 May 2;15(5):e1002566. doi: 10.1371/journal.pmed.1002566 (PMC5931494; doi:10.1371/journal.pmed.1002566)
Supplement: S2 Table — (DOCX) [file pmed.1002566.s010.docx]

**S2 Table: Studies included in the meta-analysis of the association of the 2-SNP genetic score influencing plasma 25(OH)D concentration with risk of diabetes**

| **Study (Supplementary reference)** | **Latitude** | **No. of**  **Cases** | **No. of**  **Controls** |
| --- | --- | --- | --- |
| ***Northern latitude*** |  |  |  |
| Cambridgeshire Case-Control Study (CCCS) ([S-1](#_ENREF_1), [S-2](#_ENREF_2)) | 52 | 538 | 520 |
| ADDITION-Ely ([S-2](#_ENREF_2), [S-3](#_ENREF_3)) | 52 | 838 | 1474 |
| Norfolk-Diabetes (S-[2](#_ENREF_2), S-[4](#_ENREF_4)) | 52 | 5434 | 6059 |
| DIAGRAM ([S-5](#_ENREF_5)) | 55 | 9580 | 53810 |
| Copenhagen ([S-6](#_ENREF_6)) | 55 | 5037 | 91,386 |
| UKB ([S-7](#_ENREF_7)) | 52 | 6234 | 106,104 |
| ***Southern latitude*** |  |  |  |
| EPIC-InterAct-Metabochip ([S-2](#_ENREF_2), [S-8](#_ENREF_8), [S-9](#_ENREF_9)) | 50 | 3518 | 5870 |
| EPIC-InterAct-GWAS ([S-2](#_ENREF_2), [S-8](#_ENREF_8), [S-9](#_ENREF_9)) | 50 | 4653 | 4690 |
| CKB ([S-10](#_ENREF_10)) | 32 | 5565 | 76,871 |
| T2D Exome Consortium ([S-11](#_ENREF_11)) | 34 | 16,915 | 23,808 |
| **Total** |  | **58,312** | **370,592** |

**Supplementary References for studies shown in S2 Table**

1. Halsall DJ, McFarlane I, Luan J, Cox TM, Wareham NJ. Typical type 2 diabetes mellitus and HFE gene mutations: a population-based case - control study. Hum Mol Genet. 2003;12: 1361-1365.
2. Ye Z, Sharp SJ, Burgess S, Scott RA, Imamura F, InterAct Consortium, et al. Association between circulating 25-hydroxyvitamin D and incident type 2 diabetes: a mendelian randomisation study. Lancet Diabetes Endocrinol. 2015;3: 35-42.
3. Echouffo-Tcheugui JB, Simmons RK, Williams KM, Barling RS, Prevost AT, Kinmonth AL, et al. The ADDITION-Cambridge trial protocol: a cluster – randomised controlled trial of screening for type 2 diabetes and intensive treatment for screen-detected patients. BMC Public Health. 2009;9: 136.
4. Forouhi NG, Ye Z, Rickard AP, Khaw KT, Luben R, Langenberg C, et al. Circulating 25-hydroxyvitamin D concentration and the risk of type 2 diabetes: results from the European Prospective Investigation into Cancer (EPIC)-Norfolk cohort and updated meta-analysis of prospective studies. Diabetologia. 2012;55: 2173-2182.
5. Diabetes Genetics Replication and Meta-analysis (DIAGRAM). (Cited 2 March 2017). Available from: <http://diagram-consortium.org/about.html>.
6. Afzal S, Brøndum-Jacobsen P, Bojesen SE, Nordestgaard BG. Vitamin D concentration, obesity, and risk of diabetes: a mendelian randomisation study. Lancet Diabetes Endocrinol. 2014;2: 298-306.
7. UK Biobank. (Cited 2 March 2017). Available from: <https://www.ukbiobank.ac.uk/resources/>.
8. European Prospective Investigation into Cancer and Nutrition (EPIC) -Interact. (Cited 2 March 2017). Available from: <http://www.mrc-epid.cam.ac.uk/research/studies/interact/>.
9. The InterAct Consortium. Design and cohort description of the InterAct Project: an examination of the interaction of genetic and lifestyle factors on the incidence of type 2 diabetes in the EPIC Study. Diabetologia. 2011;54: 2272-2282.
10. China Kadoorie Biobank Collaborative Group. (Cited 2 March 2017). Available from: <http://www.ckbiobank.org/site/>.
11. Type 2 Diabetes Genetic Exploration by Next-generation sequencing in multi-Ethnic Samples (T2D-GENES). (Cited 2 March 2017). Available from: <https://t2d-genes.sph.umich.edu/>.
