# Supplementary material for: Association of vitamin D with risk of type 2 diabetes: A Mendelian randomisation study in European and Chinese adults
Source: PLoS Med. 2018 May 2;15(5):e1002566. doi: 10.1371/journal.pmed.1002566 (PMC5931494; doi:10.1371/journal.pmed.1002566)
Supplement: S3 Table — (DOCX) [file pmed.1002566.s011.docx]

**S3 Table: Plasma 25(OH)D concentration by relevant confounders in CKB**

| **Characteristic** | **No. of participants** | **Mean (SD) 25(OH)D concentration, nmol/L*** |
| --- | --- | --- |
| **Age, years** |  |  |
| <60 | 9032 | 61.7 (22.53) |
| 60+ | 4037 | 63.5 (27.17) |
|  |  |  |
| **Sex** |  |  |
| Male | 6666 | 67.6 (17.15) |
| Female | 6403 | 56.6 (17.15) |
|  |  |  |
| **Season** |  |  |
| Winter | 3269 | 57.4 (16.79) |
| Spring/Autumn | 6713 | 61.8 (16.79) |
| Summer | 3087 | 68.4 (16.76) |
|  |  |  |
| **Physical activity, MET-h/day** | | |
| Bottom tertile (≤11.2) | 4356 | 61.8 (18.34) |
| Middle tertile (11.3 - 24) | 4361 | 63.2 (17.23) |
| Top tertile (>24) | 4352 | 61.7 (18.60) |
|  |  |  |
| **Fat percent, %** | |  |
| Bottom tertile (≤22.6) | 4362 | 64.7 (19.75) |
| Middle tertile (22.7 - 30.3) | 4370 | 61.9 (17.12) |
| Top tertile (>30.3) | 4337 | 60.1 (19.43) |
| **ALL** | **13,069** | **62.2 (20.31)** |

*All values are adjusted for age, sex, area and season as appropriate
