# Supplementary material for: Association of vitamin D with risk of type 2 diabetes: A Mendelian randomisation study in European and Chinese adults
Source: PLoS Med. 2018 May 2;15(5):e1002566. doi: 10.1371/journal.pmed.1002566 (PMC5931494; doi:10.1371/journal.pmed.1002566)
Supplement: S4 Table — (DOCX) [file pmed.1002566.s012.docx]

**S4 Table: Comparison of observational associations of plasma 25(OH)D concentration with risk of diabetes in the CKB study with the meta-analysis of prospective European cohorts**

| **25(OH)D Group** | **Type of cases** | **Adjustments** | **Diabetes cases** | **Diabetes controls** | **Odds ratio (95% CI) per  25 nmol/L higher 25(OH)D concentrations** | **Chi-square** | **P-value** |
| --- | --- | --- | --- | --- | --- | --- | --- |
| CKB | Incident cases | Latitude, sex, systolic blood pressure, total physical activity, fat percent, season and age | 979 | 12,090 | 0.91 (0.82, 1.00) | 3.7 | 5 x 10^-2^ |
| Meta-analysis of European prospective cohort studies* | Incident cases | Age, sex, season, physical activity, measures of adiposity, blood pressure, smoking, lipids, education, dietary intake, prior history of CVD or diabetes | 8492 | 89,698 | 0.83 (0.79, 0.86) | 70.4 | 7 x 10^-19^ |
| Chinese and European populations combined |  |  | 9471 | 101,788 | 0.84 (0.81, 0.88) | 71.5 | 4 x 10^-18^ |

*Not all of these factors were adjusted for in every cohort study included in the meta-analysis (Ye et al. Lancet Diabetes Endocrinol 2015;3: 35–42).
