# Supplementary material for: Association of vitamin D with risk of type 2 diabetes: A Mendelian randomisation study in European and Chinese adults
Source: PLoS Med. 2018 May 2;15(5):e1002566. doi: 10.1371/journal.pmed.1002566 (PMC5931494; doi:10.1371/journal.pmed.1002566)
Supplement: S5 Table — (DOCX) [file pmed.1002566.s013.docx]

**S5 Table: Allele frequency of individual SNPs and effect of genetic scores on mean plasma 25(OH)D concentration using the synthesis SNPs and all four 25(OH)D SNPs, by latitude in CKB**

| **Area^1^** | **Latitude** | **Mean 25(OH)D, nmol/L** | **rs12785878 (DHCR7) EAF^2^** | |  | **rs10741657 (CYP2R1) EAF^2^** | **Mean (SE)  effects of GRS (DHCR7+CYP2R1)^3^ on 25(OH)D** |  | **rs6013897 (CYP24A1) EAF^2^** | **rs2285878 (GC/DBP) EAF^2^** | **Mean (SE) effects of GRS (All 4 SNPs)^4^ on 25 (OH)D** |
| --- | --- | --- | --- | --- | --- | --- | --- | --- | --- | --- | --- |
| Harbin (urban) | 46 | 49 | | 0.51 |  | 0.39 | 0.68 (0.89) |  | 0.84 | 0.68 | 1.47 (0.75) |
| Qingdao (urban) | 36 | 57 | | 0.47 |  | 0.38 | 3.32 (1.79) |  | 0.85 | 0.67 | 3.89 (1.51) |
| Henan (rural) | 35 | 58 | | 0.51 |  | 0.37 | 1.79 (0.97) |  | 0.84 | 0.69 | 2.50 (0.81) |
| Gansu (rural) | 35 | 53 | | 0.54 |  | 0.39 | 3.76 (0.79) |  | 0.83 | 0.69 | 3.63 (0.65) |
| Suzhou (urban) | 31 | 67 | | 0.43 |  | 0.36 | 2.50 (2.36) |  | 0.84 | 0.68 | 3.41 (1.83) |
| Sichuan (rural) | 31 | 65 | | 0.45 |  | 0.35 | 3.81 (1.41) |  | 0.83 | 0.70 | 4.98 (1.15) |
| Zhejiang (rural) | 31 | 68 | | 0.46 |  | 0.36 | 3.82 (1.60) |  | 0.84 | 0.67 | 3.27 (1.32) |
| Hunan (rural) | 28 | 73 | | 0.44 |  | 0.34 | 3.92 (1.07) |  | 0.83 | 0.71 | 5.55 (0.86) |
| Liuzhou (urban) | 24 | 70 | | 0.38 |  | 0.30 | 3.68 (1.54) |  | 0.84 | 0.75 | 4.91 (1.24) |
| Haikou (urban) | 20 | 90 | | 0.36 |  | 0.30 | 9.55 (4.03) |  | 0.84 | 0.75 | 6.32 (2.81) |
| **Total** | **-** | **62** | | **0.46** |  | **0.36** | **3.15 (0.41)** |  | **0.84** | **0.70** | **3.86 (0.34)** |
| **P for trend** |  | **3.3 x 10^-39^** | |  |  |  | **0.03** |  |  |  | **0.06** |

^1^Areas are ordered by latitude from North to South.

^2^ EAF: effect allele frequency based on frequency of 25(OH)D increasing allele. SNP rs10741657 had 12 missing genotypes, rs12785878 had 16 missing genotypes, rs6013897 had 68 missing genotypes, rs22826279 had 168 missing genotypes.

^3^Genetic score is weighted based on the per allele association of 25(OH)D synthesis SNPs rs12785878 and rs10741657 with 25(OH)D concentrations.

^4^Genetic score is weighted based on the per allele association of all four 25(OH)D SNPs rs12785878, rs10741657, rs6013897 and rs2282679 with 25(OH)D concentrations.
